# Trend of hoverboard related injuries at a pediatric emergency department

**DOI:** 10.1186/s13052-022-01227-4

**Published:** 2022-04-01

**Authors:** Valentina Ferro, Raffaella Nacca, Elena Boccuzzi, Tatiana Federici, Chiara Ossella, Alessandra Merenda, Renato Maria Toniolo, Anna Maria Musolino, Antonino Reale, Umberto Raucci

**Affiliations:** 1 Pediatric Emergency, Department of Emergency and General Pediatrics, Bambino Gesù Children’s Hospital, IRCCS, Rome, Italy; 2 Emergency Department, Bambino Gesù Children Hospital, IRCCS, Palidoro, Italy; 3 U.O.C. of Orthopedics and Traumatology, Bambino Gesù Children’s Hospital, IRCCS, Rome, Italy

**Keywords:** Injury, Hoverboard, Safety, Prevention, Child, Emergency

## Abstract

**Introduction:**

Understanding how the use of hoverboards (HBs) can affect a child’s safety is crucial. We describe the characteristics of HB related injuries and provide key messages about child prevention when using these leisure devices.

**Methods:**

This was a retrospective study at an emergency department (ED) of a level-III-trauma center from 2016 to 2019. We tested the differences in children presenting for injury associated with HBs between 2016-2017 and 2018-2019 to better describe the temporal trend of the phenomenon.

**Results:**

The rate of *Injury associated with HBs / Total injury per 1,000* increased from 0.84 in 2016 to 7.7 in 2017, and then there was a gradual decline. The likelihood of injury was more common in younger children, increasing by 17% with decreasing age in 2018-2019 compared with 2016-2017 (OR: 0.83; 95%CI: 0.71-0.97; *p* = 0.021). The occurrence of injury in the April-June period was over twice as common in 2018-2019 (OR: 2.05; 95%CI: 1.0-2.05; *p* = 0.05). Patients were over 4 times more likely to have injured the lower extremity during the 2018-2019 period rather than other body regions (OR: 4.58; 95%CI: 1.23-4.58; *p* = 0.02). The odds of the indoor injury were more than twice as high in 2018-2019 (OR: 2.04; 95%CI: 1.077-2.04; *p* = 0.03).

**Conclusion:**

Despite a decrease in the frequency of HB related injuries after 2017, during the 2018-2019 period, the younger the children, the more they were exposed to injury risk, in addition to a greater occurrence of indoor injuries from HBs compared with 2016-2017. The enhancement of preventive measures is necessary to ensure child safety when using HBs.

## Introduction

Although hoverboards (HBs) as leisure devices have many benefits such as the easy learning and portability, the reasonable cost, environmentally friendly, in addition to being practical, useful, and a fun device that delights both young people and adults alike. However, they also present shortfalls of which safety concern is the most important. The principal safety concern is the injury from a fall. In this perspective, HBs are a potential threat to the safety of the pediatric population. First, the speed can represent a warring danger for both children and teenagers. In fact, the HB can reach a top speed of 5-15 miles per hour [1] and this might be exciting and attractive for teenagers who often strive to perform more challenging and daring acts; this behavior is known as “risk-taking,” [2] and therefore, they are prone to uncontrolled speed. Likewise, younger children are vulnerable to injury due to their physiologically immature motor skills [3] that can compromise the balance and the agility of tilting the pad and of controlling the speed and the direction of travel. This consideration is strengthened by the fact that pediatric patients are more at risk for HBs related injuries than adults[4].

Our study derives from the consideration that a specific European regulation has been made for the use of the bicycle [5] but not for other leisure wheeled devices such as HBs. Our aim was to describe the trend, the epidemiology, and characteristics of HBs related injuries at an Italian pediatric emergency department (ED) of a tertiary hospital in order to offer suggestions about child prevention when using these devices.

## Methods

This was a retrospective study conducted at the ED of the level-III-trauma center of a Children’s Hospital in Rome during two consecutive periods, 2016-2017 and 2018-2019. We chose to compare these two periods to better describe the temporal trend of the phenomenon.

We enrolled children aged to < 18 years admitted to the ED with a diagnosis of injuries associated with the usage of an HB. We collected demographic data and injury characteristics for all patients from electronic chart at ED, GIPSE (Gestione Informazione Pronto Soccorso Emergenza -Informational Management for First Aid and Emergency). The extracted data included age, sex, date of injury, location of injury (indoors versus outdoors), priority of admission to ED, injury site, type of injury, need of recovery, follow up after discharge, and need of treatment (conservative vs surgical management).

The age was categorized into 3 groups based on the child development: preschool age (3-5 years), middle childhood (6-9 years), early and late adolescence (≥ 10 years).

At the admission of each child in our ED, the priority of assessment was assigned in accordance with clinical condition severity, in line with the regional guidelines of Lazio: the need of immediate-intermediate medical care versus low-non urgent medical care [6].

The injured body region was classified into the following groups: face/trunk; head/neck; upper extremity, and lower extremity. The upper extremity group included the upper and lower arm, shoulder, elbow, wrist, hand, and fingers. The lower extremity category included the upper and lower leg, knee, ankle, foot, and toes. The face and trunk category included the eyes, nose, ears, mouth with lips, tongue and teeth, the upper and lower trunk and pubic region. The neck and head were reported separately. The time of presentation in ED was categorized into early (< 24 hours from the time of injury) and late (≥ 24 hours from the time of injury). The injury place was distinguished into indoors and outdoors.

A discharge diagnosis (type of injury) performed by an ED physician was classified into diagnostic categories as follows: fracture, contusion, laceration/abrasion, parenchymal injury, and concussion.

We used two epidemiological indicators to describe the temporal trend of injuries associated with IBs*: Injury associated with HBs / Total injury per 1,000* and *Injury associated with HBs / ED visits per 1,000*.

We also compared the percentage of injuries by month and year, and we evaluated the popularity of HBs using Google Trends as a tool that allows to access research trends on the internet network. In fact, we entered “Hoverboard” as a keyword to explore trending searches on this topic in the Lazio region during the period 2016-2019. This parameter was reported as a score 0 to 100, where 100 means a very highly researched item. The study was approved by the Ethics Committee of our Children’s Hospital according to the Declaration of Helsinki (as revised in Seoul, Korea, October 2008). The number protocol was 1793_OPBG_2019.

### Data analysis

A Statistical analysis was performed using the software STATA/IC 14.2 version 2017. Categorical variables were reported as proportion and percentage, and continuous variables were described as mean and standard deviation (SD). We tested the differences between children presenting to the ED for injury associated with HBs in biennium 2016-2017 and the most recent biennium 2018-2019. We used the two-sample proportion test to know whether the groups differ significantly on some single (categorical) characteristic while we used the two-sample t-test to determine if the two groups are equal for continuous variables. The percentage differences and the mean differences with 95% confidence intervals (CIs) were reported. In addition, a logistic multivariable regression analysis was carried out to explore variables associated with the most recent biennium 2018-2019 compared with biennium 2016-2017. We considered a 2-tailed *p* value less than 0.05 to be significant.

## Results

**Fig. 1:**
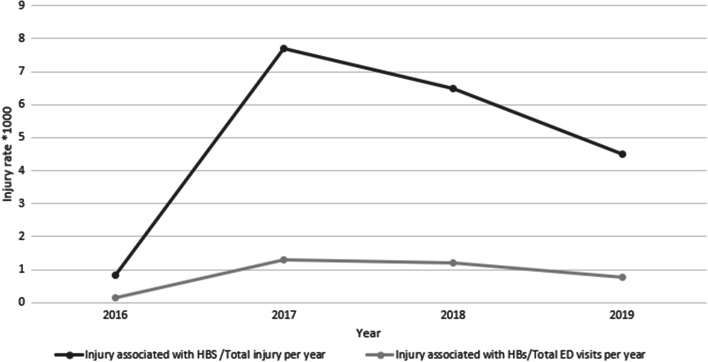
Temporal trend of injuries associated with hoverboards in a hospital Emergency Department (2016-2019)

**Fig. 2:**
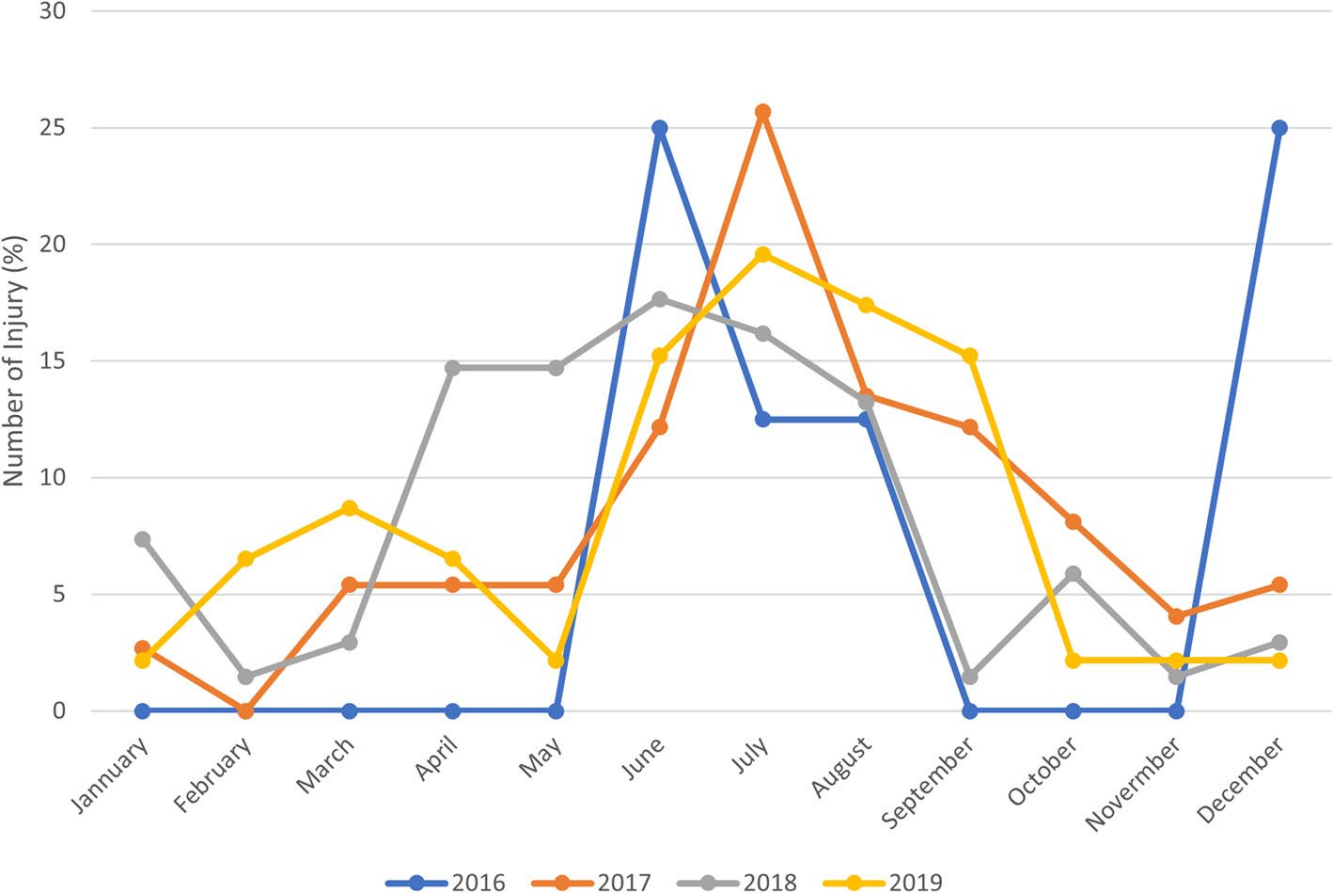
The occurrence of injuries associated with HBs monthly per year

**Fig. 3:**
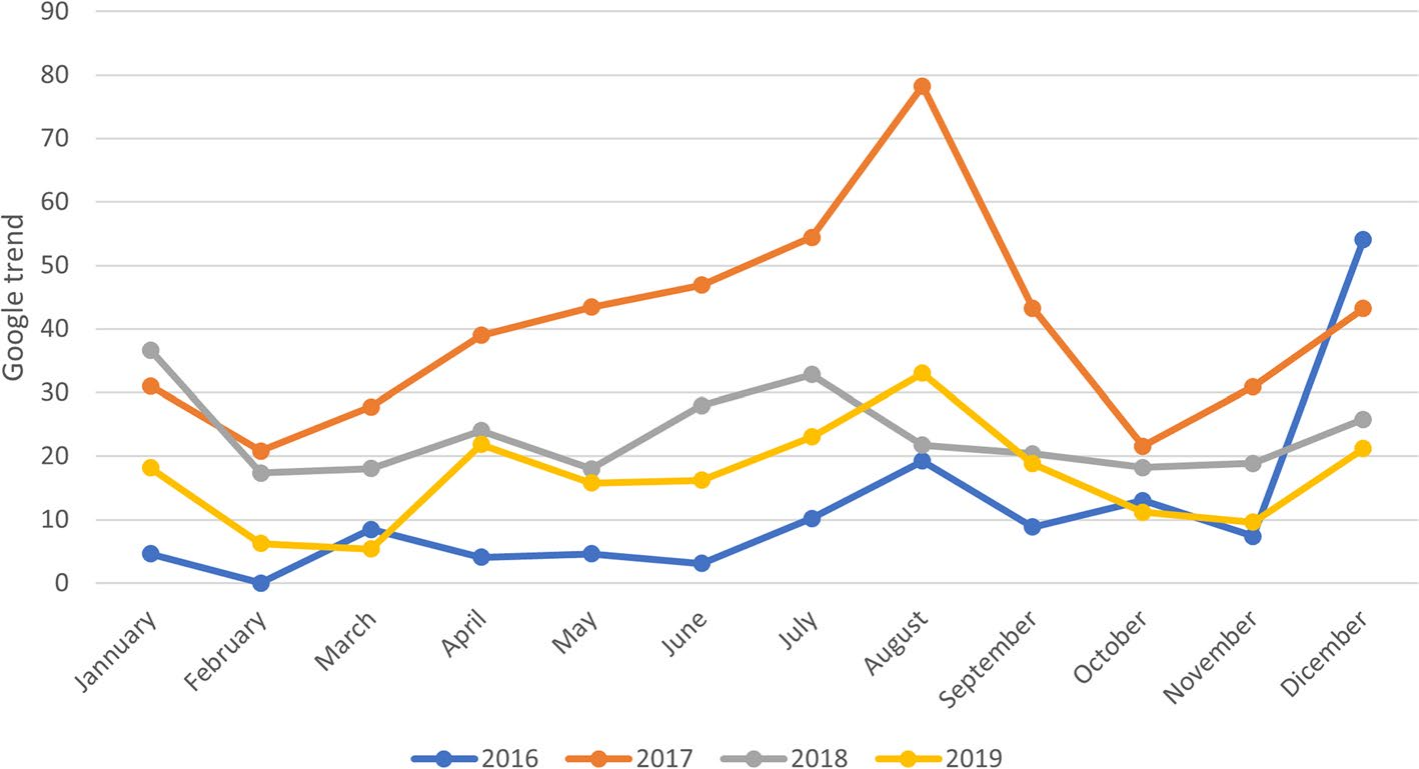
The popularity of HBs in region Lazio from 2016 to 2019 extrapolated by Google Trends

During our study period, we estimated an average of 9,985 injuries annually, and an average of 57,138 visits annually at the ED in our institution. The occurrence of HBs related injuries varied over the years, accounting for 8 cases in 2016, reaching a spike in 2017 (78 cases) and decreasing progressively to count 46 cases in 2019. We reported an increased rate of *Injury associated with HBs / Total injury per 1,000* from 0.84 in 2016 to 7.7 in 2017 and then a following and gradual decline (Fig. 1). Likewise, we also described an increase in the rate of *Injury associated with IBs / ED visits per 1,000* accounting for 0.14 in 2016 and 1.3 in 2017 and then a progressive decrease (Fig. 1). Analyzed in terms of monthly occurrence per year, the distribution of the injury associated with HBs was variable, even if the peak of injury was mostly concentrated in spring and summer. In 2016 we also reported a great peak before December (Fig. 2). This temporal relationship of injuries by month almost reflected the popularity of the HBs extrapolated by Google Trends during the same period in Lazio (Fig. 3). In fact, the interest expressed by users of the web on HBs showed that it obtained a peak during the May-October period over years, and a second, but minor peak before December in 2016 and 2017.

In Table 1, we reported the baseline characteristic of HBs related injuries during the entire study period.

**Table 1:** Baseline characteristics of injuries associated with HBs in children admitted to ED during period 2016-2019

**Characteristics196**	**No**	**%**	**95% CI**	
**Age group**				
Preschool child (3-5yrs)	9	4.59	1.6	7.5
Middle childhood (6-9 yrs)	96	48.98	41.9	56
Adolescent (10-18 yrs)	91	46.43	39.4	53.5
**Sex**				
Female	73	37.24	30.4	44.1
Male	123	62.76	56	69.6
**Injury seasonal period**				
January-March	22	11.22	6.8	15,7
April-June	62	31.63	25.1	38.2
July-September	87	44.39	37.4	51.4
October-December	25	12.76	8	17.5
**Injury place**				
Indoor injury	110	56.12	48.87	63.18
Outdoor injury	86	43.88	36.82	51.13
**Admission to ED**				
Direct	177	90.31	86.13	94.48
Transferred	19	9.69	5.94	14.72
**Priority of admission to ED**				
Immediate/intermediate care needed	60	30.61	24.1	37.12
Not urgent care needed	136	69.39	62.42	75.76
**Injury site**				
Face/Trunk	18	9.18	5.10	13.26
Head/Neck	20	10.20	5.93	14.48
Upper extremity	142	72.45	66.14	78.76
Lower extremity	19	9.69	5.52	13.80
**Type of injury**				
Fracture	129	65.82	59.12	72.52
Contusion	51	26.02	20.03	32.75
Laceration/abrasion	10	5.10	2.47	8.21
Dislocation/Sprain	3	1.53	-0.20	3.26
Parenchymal injury	1	0.51	0.01	2.81
Concussion	6	3.06	0.63	5.49
**Need of recovery**	50	25.51	10.35	31.67
**Need of follow up**	142	72.45	65.63	78.58
**Need of treatment**	140	71.43	64.56	77.64
Conservative management	97	49.49	42.29	56.71
Surgical management	43	21.94	16.36	28.39

The frequency of injury was 4.59% in preschoolers, 48.98% in middle childhood, and 46.43% in adolescence, respectively. Most of these injuries occurred during the July-September period (44.39%) and surprisingly, indoor injuries were more common than those outdoors (56.12% versus 43.88%). The most common injured site was the upper extremity (72.45%) while the fracture was the most frequent type of injury (65.82%).

To better study the temporal trend of this phenomenon, we compared the biennium 2016-2017 with the most recent one, 2017-2019 (Table 2).

**Table 2:** Characteristic of children with hoverboard related injuries during 2 consecutive biennium (2016-2017 versus 2018-2019)

**Characteristics**	**2016-2017**		**2018-2019**		**Difference**			
	**mean**	**SD**	**mean**	**SD**	**mean**	**95% CI**		***P* value**
**Age (years)**	9.61	0.21	8.87	0.23	0.74	0.11	1.37	**0.03**
	**No.**	**%**	**No.**	**%**	**%**	**95% CI**		***P* value**
**Sex**								
• Female	27	32.93	46	40.35	7.42	-6.16	21.01	0.289
• Male	55	67.07	68	59.65				
**Injury seasonal period**								
• January-March	6	7.32	16	14.04	-6.72	-15.23	1.79	0.142
• April-June	19	23.17	43	37.72	-14.55	-27.30	-1.80	**0.03**
• July-September	42	51.22	45	39.47	11.75	-2.31	25.80	0.1
• October-December	15	18.29	10	8.77	9.52	-0.33	19.37	**0.05**
**Time of presentation**								
• Early presentation	44	53.66	47	41.23	12.43	-1.56	26.51	0.08
• Late presentation	38	46.34	67	58.77				
**Injury place**								
• Indoor	38	46.34	72	63.16	-16.82	-30.78	-2.86	**0.019**
• Outdoor	44	53.66	42	36.84				
**Priority of admission to ED**								
Immediate/intermediate care needed	28	34.15	32	28.07	6.08	-7.09	19.24	0.36
Not urgent care needed	54	65.85	82	71.93				
**Injury site**								
Face/Trunk	7	8.54	11	9.65	-1.11	-9.23	7.01	0.79
Head/Neck	8	9.76	12	10.53	-0.77	-9.31	7.77	0.86
Upper extremity	64	78.05	68	68.42	9.63	-2.74	22	0.14
Lower extremity	3	3.66	16	14.04	-10.38	-17.94	-2.82	**0.015**
**Type of injury**								
Fracture	58	70.73	71	62.28	8.45	-4.82	21.72	0.29
Contusion	17	20.73	34	29.82	-9.09	-21.24	3.05	0.15
Laceration/abrasion	1	1.22	9	7.89	-6.68	-12.17	-1.18	**0.036**
Dislocation/Sprain	2	2.44	1	0.88	1.56	-2.19	5.31	0.38
Parenchymal injury	0		1	0.88	-0.88	-2.59%	0.83	0.39
Concussion	5	6.10	1	0.88	5.22	-0.23	10.68	**0.036**
**Need of recovery**	21	25.61	28	24.56	1.05	-11.27	13.36	0.87
**Need of follow up**	59	71.95	83	72.81	-0.86	-13.55	11.84	0.89
**Need of treatment**	63	76.83	77	67.54	0.29	-3.26	21.83	0.16
Conservative management	44	69.84	53	68.83	1.01	-14.33	16.35	0.9
Surgical management	19	30.16	24	31.17	-1.01	-16.35	14.33	0.9

For demographic characteristics, we noted that children with HBs related injuries in 2018-2019 were significantly younger (9.61 ± 0.21 SD versus 8.87 ± 0.23; *p* = 0.03), but no significant difference for sex was demonstrated. From a point of a seasonal view, the rate of injury was significantly greater in the April-June period in 2018-2019 than 2016-2017 (23.17% versus 32.72%; *p* = 0.03), and in October-December in 2016-2017 (18.29 %versus 8.77%; *p* = 0.05). We reported that a significant number of HBs were being used indoors in 2018-2019 rather than 2016-2017 (63.16% versus 46.34; *p* = 0.019).

Analyzing the injury body region, there was a significant occurrence of injured lower extremities in the 2018-2019 period**,** more than the 2016-2017 period (3.66% versus 14.04%; *p* = 0.015). We observed no significant difference between the two periods on distribution of the fracture that represented the most common type of injury, but we observed a significant difference for laceration/abrasion that occurred more frequently in the period 2018-2019 (1.22% versus 7.89%; *p* = 0.036) and for concussion, it was more common in the period 2016-2017 (6.10% versus 0.88%; *p* = 0.036). No significant difference was described for need of recovery, follow up, and need of treatment between the two periods.

In Table 3 we explored the variables associated with the most recent biennium 2018-2019 compared with the previous two-year period 2016-2017 by logistic regression analysis. We noted that the likelihood of injury related to HBs was more common in younger children; in particular, it increased by 17% with decreasing age in 2018-2019 compared with 2016-2017 (Odd Ratio-OR-: 0.83; 95%CI 0.71-0.97; *p* = 0.021). The occurrence of injury in the April-June period was over twice as common in 2018-2019 than 2016-2017 (OR: 2.05; 95%CI 1.0-2.05; *p* = 0.05). Patients were over 4 times more likely to have injured the lower extremity during the 2018-2019 period rather than other body regions compared with the 2016-2017 period (OR: 4.58; 95%CI 1.23-4.58; *p* = 0.02). While the odds that a child presented concussion were lower in 2018-2019 than 2016-2017 (OR: 0.09; 95%CI: 0.01-0.09; *p =* 0.05). Finally, the odds of the indoor injury were more than twice as high in the 2018-2019 biennium (OR: 2.04; 95%CI: 1.077-2.04; *p* = 0.03)

**Table 3:** Logistic regression analysis exploring variables associated with the most recent biennium 2018-2019 compared with biennium 2016-2017

**Characteristics**	**OR**	**SD**	**z**	***P***	**95% CI**	
					**Lower**	**Upper**
**Age (years)**	0.83	0.07	-2.31	**0.021**	0.71	0.97
**Female vs male**	1.63	0.56	1.44	0.15	0.84	3.18
**Injury seasonal period** April-June	2.05	0.76	1.95	**0.05**	1.00	2.05
**Injury seasonal period** October-December	0.56	0.28	-1.17	0.24	0.21	0.56
**Early time of presentation**	1.69	0.55	1.61	0.11	0.89	1.69
**Injured lower extremity**	4.58	3.07	2.27	**0.02**	1.23	4.58
**Laceration/abrasion**	3.36	3.73	1.09	0.27	0.38	3.36
**Concussion**	0.09	0.11	-1.97	**0.05**	0.01	0.09
**Indoors vs outdoors**	2.04	0.67	2.16	**0.03**	1.07	2.04

## Discussion

To the best of our knowledge, our study is, in European pediatric literature, the first observational ED-based research analyzing the frequency, the temporal trend, and the pattern of the injuries associated with HBs in pediatric patients in an effort to recognize the principal criticalities regarding the usage of these recreational devices in the pediatric population.

We reported an increased rate of *Injury associated with HBs / Total injury per 1,000*from 0.8 in 2016 to 7.7 in 2017, then a gradual decline over the following year. Compared with a previous American study, in 2015 there was an average 208% increase in the rate of this type of injury compared with any of the previous 4 years [7]. Probably, this temporal difference with our data is due to a delay of 2 years of the introduction of HBs in the Italian consumer market compared with the boom in the US. Overall, our data revealed a positive trend because after a peak in 2017, the rate of injuries declined in 2018 and 2019. If we consider the interest of users online in HBs by Google Trends, we noticed that the popularity in the Lazio region, after a peak during 2017, decreased over time. Therefore, the reduction of HBs related injuries was, in all probability, due to a decrease in popularity after 2017, taking a back seat in the market of the electric mobility on two wheels because of the success of other, more novel and sophisticated paradigms such as the electric scooter that is now the most popular electric wheelers in European cities [8].

We observed that the frequency of HB related injuries was variable, but generally the peak of injuries was concentrated in spring and summer. This distribution not only followed the popularity trend but relies on other factors such as the seasonality and weather conditions that can influence physical activities. In fact, a systematic review reported that a seasonal effect is present as the levels of physical activity appear to be highest in spring and summer [9]. Numerous attributes might affect physical activity behaviors such as temperature, precipitation, and daylight hours [9]. In fact, with a longer day length in spring, undoubtedly for the child**,**the recreational activities increased, and there is a greater desire to run and play [10]. Instead, in 2016 we reported a peak just before December, and this data might be explained by the novel recreational product just introduced in the Italian market that year and in coincidence with Christmas sales resulting in one of the most popular and attractive gifts [4, 7, 11, 12].

We noticed that younger children were at a higher risk of HB related injuries in 2018-2019 in comparison with 2016-2017. In the literature, many studies report that pediatric patients are more at risk of injury than adults [3, 4, 7, 13] and generally, the use of HBs and the injuries associated with them are more common in the adolescent population [13–16].

It was surprising how a significant proportion of HBs were being used indoors, and this data has also been described in other studies [13, 16].Moreover, in 2018-2019 we registered a significant, greater proportion of injuries occurring indoors than in 2016-2017. This finding of extreme concern might be analyzed form different points of view. Generally, older children present a higher rate of outdoor injuries [17]. We reported a higher risk of indoor injury occurring in 2018-2019 because injuries occurred more commonly in younger children who tend to play indoors. A higher proportion of indoor injuries in the last years might also mirror the considerable evidence that the opportunities to play outdoors have steadily declined across the last generations, and the leisure lives of children appear to be moving indoors [18–21]. Some researchers have stated that social influences on parents can have influenced possibilities in outdoor play engagement of children [22–25].

However, we assume that the principal cause of this phenomenon is a shortage of an adequate awareness and education of parents and other caregiver figures about the risks related to the usage of these devices. Parents should be educated on the appropriate location for the use of this leisure device for their children, on the safety equipment that should be used at all times while on board HBs, and on the enrollment of the child in lessons. Safety policies of the governments are also important to promote preventive programs for modifying behavior. In Italy the legislative decree n° 229, 4 June 2019 [26] opens to the experimentation on the devices of the electric micro-mobility including HBs, but the age limit is a factor depending just on common sense because the current legislation does not consider this issue. The best usage of the HB has been conceived for adults for two reasons. First, children are physiologically at risk for falls because of less mature development in coordination, balance motor strength, along with their higher center of gravity [3]. The second reason is one of responsibility. In fact, Italian law establishes that in a case of a child caught in breach of the usage regulations, the penalty will be issued to the parents. The penalty is applied not because the device has been ridden by a child, but because the device circulates on a forbidden area such as a street (regulation n° 190 of the Italian Highway Code) [27]. In Italy, the driver’s “AM” license can be obtained upon reaching 14 years of age and allows riding two wheeled vehicles with a maximum engine displacement of 50 cm^3^, a maximum power of 4 Kw, and a maximum speed of 45 km / h. This age limit might be a compromise solution.

Analyzing the site of the injury, the upper extremity was the most common region of the body to be affected, and the fracture was the most common type of injury. These findings were replicated by other studies [4, 11, 12, 14, 16, 28]. Some of these studies also detailed an increase in the wrist and forearm injuries, specifically fractures. Surprisingly, patients were almost 5 times more likely to have injured the lower extremity during the 2018-2019 period than other body regions compared with the 2016-2017 period, when, generally, younger children appear to be pre-disposed to injuries of the upper extremity [29]. We are not able to explain this data if we consider that the likelihood of injury was higher in younger children in the last biennium. Probably, we did not consider the potential confounders of the relationship between body region and injury, such as the body mass index, bone mass and bone minerality density, stature, inadequate nutrition, cognitive development, use of medication, mechanism of injury (fall or collision), fall from stationary or moving HB, fall backwards, forwards or sideways, and different kinetic energy depending on riding along a downhill or flat pathway.

Our study has some limitations. The nature of a retrospective study makes it subjected to shortcomings associated with this type of analysis: documentation omissions, missed relevant data, selection bias. The single site of our study may affect the generalizability of the results, although in the Lazio region, there are only 2 major pediatric trauma hospitals in a 17,242 km^2^ wide region. GIPSE lacks a specific code for HBs; therefore, the diagnosis of HB related injuries was extrapolated from a narrative mention in the clinical history of the injury as reported, and some data might be missed.

## Conclusion

After the initial enthusiasm when HBs captured the Italian market of the leisure devices, their popularity and injuries declined in the last two years 2018-2019. However, a lot of critical points and challenges remain unsolved about HB related injuries in the pediatric population.

The most important results showed that in the last biennium, the younger the children, the more they were exposed to injury risk, in addition to greater occurrence of indoor injuries from HBs compared with the previous biennium. The acquisition of fundamental motor, cognitive, and perceptual skills are the basis in developing the ability of engaging in driving a HB that requires balance, coordination, and perception of the risk conditions. Therefore, the premature use of HBs in children can result in injury. The enhancement of preventive measures is necessary to ensure the safety in younger children and for this we suggest, in addition to a rigorous adherence with the protective equipment during driving HB, a restriction from riding HBs at least in children younger than 14 years, and we encourage parents and children to avoid their indoor use.

## Data Availability

The datasets analyzed during the current study are available from the corresponding author on reasonable request.

## Abbreviations

CI: Confidence Interval
ED: Emergency Department
GIPSE: Gestione Informazione Pronto Soccorso Emergenza (Informational Management for First Aid and Emergency)
HB: Hoverboard
OR: Odd Ratio
SD: Standard Deviation
